# Multivalent antibody-based conjugates as new tools for tailored modulation of G protein–coupled receptors

**DOI:** 10.1111/bph.70519

**Published:** 2026-06-01

**Authors:** Shivani Sachdev, Ross W. Cheloha

**Affiliations:** 1 Laboratory of Bioorganic Chemistry, Bethesda, Maryland, USA; 2 National Institutes of Diabetes, Digestive and Kidney Diseases, Bethesda, Maryland, USA; 3 National Institutes of Health, Bethesda, Maryland, USA; 4 Advanced Centre for Treatment, Research and Education in Cancer (ACTREC), Tata Memorial Centre, Navi Mumbai, Maharashtra, India; 5 Training School Complex, Homi Bhabha National Institute, Mumbai, India

**Keywords:** antibody engineering, bioconjugation, bitopic ligands, G protein-coupled receptors, nanobody–ligand conjugates, pathway-selective signalling

## Abstract

The G protein–coupled receptor (GPCR) superfamily consists of the most common targets of approved drugs. Targeting GPCRs offers appealing avenues for therapeutic development. Antibodies and their fragments, such as single-domain antibodies (VHHs or nanobodies), have emerged as useful alternatives to small molecule pharmacophores as building blocks in multivalent conjugates. Antibodies and nanobodies bind targets through a binding surface that offers high affinity and specificity while facilitating engagement of GPCR epitopes not easily amenable to small molecule binding. Nanobodies, in particular, have proven valuable as tools to stabilize or selectively recognize GPCR conformations for use in structural or biosensing applications, respectively. Here, we focus on the application of antibodies and nanobodies in multivalent conjugates to address GPCR function in new ways. Such conjugates offer opportunities for highly potent and specific activation of GPCRs of interest, preferential engagement of receptor assemblies and the induction of tailored (biased) pharmacological responses. This review reports on case studies of peptide–antibody constructs, nanobody–nanobody fusions and small molecule–nanobody conjugate formats. We further discuss how emerging bioconjugation strategies and antibody engineering platforms will expand the utility of multivalent bioconjugates as next-generation therapeutic agents for GPCRs.

## 1 | INTRODUCTION

Bioactive molecules have been used to treat disease over centuries, yet the targets of these remedies remained obscure for most of human history (Liu et al., 2024). A prominent collection of such targets is the G protein–coupled receptor (GPCR) superfamily, one of the largest and most versatile protein families in the human genome. Approximately 35% of all FDA-approved therapeutic agents target GPCRs, highlighting their central role in human physiology and disease (Zhang et al., 2024). The study of GPCR function relies on ligands that selectively activate or block receptor activity. However, many endogenous ligands (proteins, peptides and small molecules) are promiscuous, with individual compounds engaging multiple GPCR subtypes and signalling pathways, complicating their utility in mechanistic studies and therapeutic development (Kleist et al., 2025; Zhang et al., 2024). One of the most enduring challenges in several areas of GPCR pharmacology is the paucity of ligands with requisite receptor subtype specificity (Lorente et al., 2025; Myslivecek, 2022). Traditionally, most characterized GPCR-targeted ligands bind to the orthosteric site, the binding pocket of the receptor that is engaged by agonists to initiate receptor activation. Such sites are typically found within the membrane embedded portion of GPCRs (Peter et al., 2024). Although orthosteric ligands remain the mainstay of GPCR pharmacology, the high conservation of these sites across GPCR families often renders efforts to identify highly selective binders challenging. Allosteric binding sites, in contrast, are generally distant from the orthosteric site and have undergone significant divergence throughout evolution. While targeting allosteric sites has proven useful in enhancing receptor subtype selectivity, progress is often constrained by limited structural and spatial information (Kenakin, 2024).

As our characterization of receptor pharmacology has expanded, additional challenges in achieving specificity have come to the forefront. For example, it is now evident that individual receptors are expressed in different tissues where their activation mediates distinct physiological responses (Daly & McGrath, 2011). Furthermore, multiple GPCRs are frequently co-expressed within an individual cell, where their signalling networks engage in dynamic interplay to modulate downstream responses (Gupte et al., 2017). Finally, GPCRs and other cell surface proteins have been shown to form oligomeric complexes (functional assemblies of two or more receptors) that tune receptor function (Ferré et al., 2020). Consideration of these hierarchical modes of GPCR organization provides a more complete understanding of cellular communication. Efforts to target these emergent levels of receptor organization offer new avenues for therapeutic selectivity. This realization has renewed interest in the development of multivalent ligands, where two or more pharmacophores are linked to simultaneously engage distinct binding sites either within the same receptor or across receptors in a complex (Huang et al., 2021). The history of linking multiple pharmacophores to create GPCR ligands dates back more than four decades (Newman et al., 2020). Recent efforts have extended and diversified this approach, as described below.

## 2 | TARGETING INDIVIDUAL RECEPTORS AND RECEPTOR COMPLEXES WITH MULTIVALENT COMPOUNDS

### 2.1 | Classification of multivalent compounds

**FIGURE 1:**
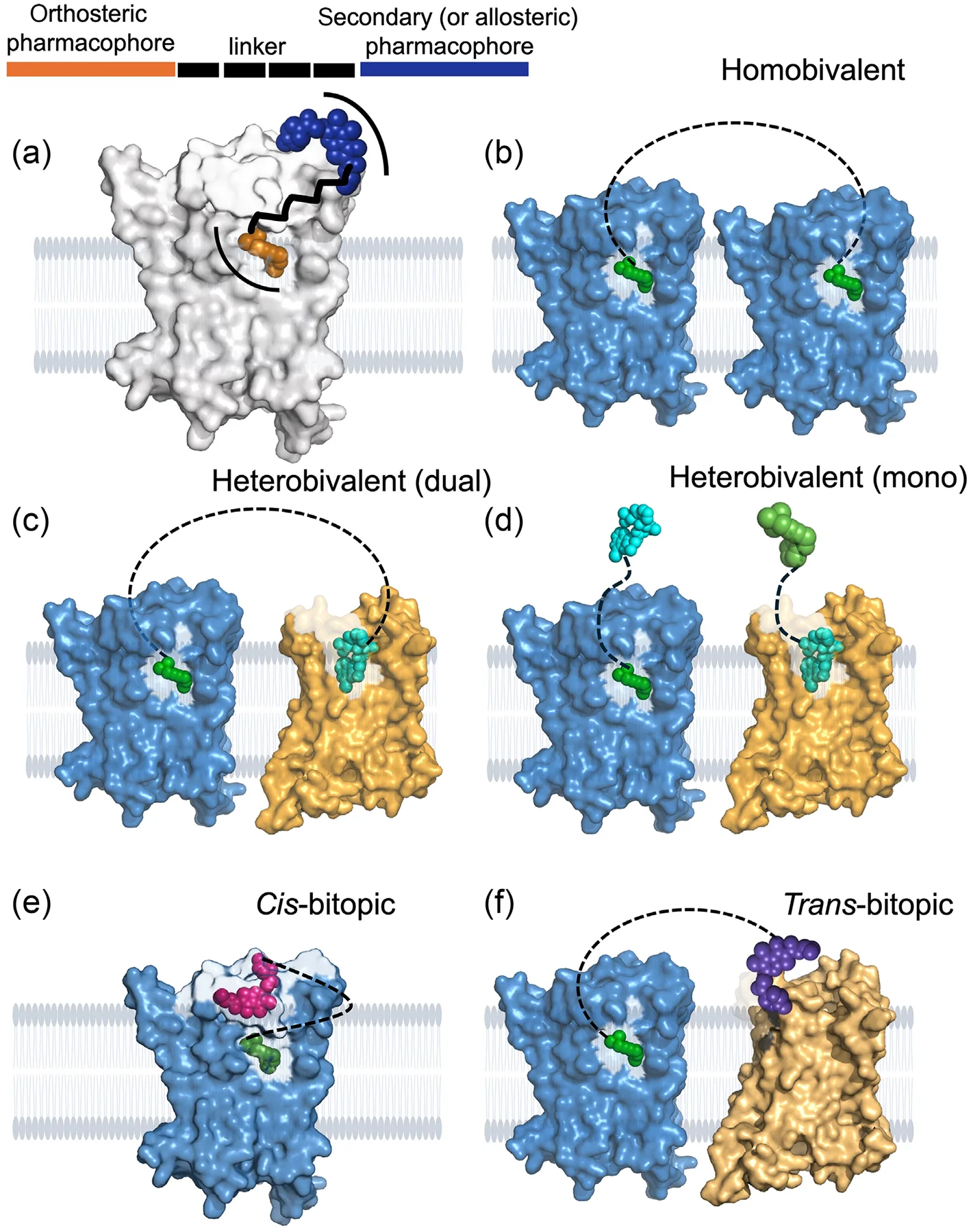
Classification of multivalent conjugates targeting GPCRs. (A) Schematic representation of a small molecule bitopic conjugate bound to a representative GPCR (M_2_ receptors). The orthosteric pharmacophore (orange) occupies the primary (orthosteric) site, while the secondary pharmacophore (blue) engages an extracellular (allosteric) site. The linker connecting the two pharmacophores is shown in black. (B) Homobivalent ligand comprised of two identical pharmacophores. (C) Heterobivalent ligands are comprised of two different pharmacophores. ‘Dual’ engagement indicates that one copy of the bivalent ligand engages two separate receptor protomers (D) ‘Mono’ engagement indicates that one copy of the bivalent ligand engages a single receptor protomer. Bitopic ligands include: (E) cis-bitopic ligand targeting the orthosteric and allosteric site within the same receptor and (F) trans-bitopic ligand engaging an orthosteric site in one receptor protomer and a secondary site in another. Structural templates used for schematic generation were M_2_ receptors (PDB 4MQT, blue) and D_3_ receptors (PDB 9F33, wheat).

Multivalent targeting constitutes a general framework for engaging receptors (such as GPCRs) with compounds that associate at more than one binding site. Most commonly, this involves the design of compounds that engage two orthosteric binding sites (bivalent ligands). Such bivalent ligands correspond to a molecule comprised of two constituent pharmacophores covalently tethered to each other via a suitable spacer (Figure 1A). If the pharmacophores are identical, such ligands are often termed homobivalent (Figure 1B); if they are different, the ligand is classified as heterobivalent (Figure 1C). Notably, certain heterobivalent compounds exhibit a ‘hook effect’ (Roy et al., 2017) where high ligand concentrations, suboptimal linker length or geometry disfavours dual engagement, leading each pharmacophore to bind its own receptor independently (Figure 1D). Careful optimization of linker design, spatial orientation of pharmacophores and consideration of receptor topology is therefore critical to avoid this outcome.

The classification of multivalent compounds is particularly important in understanding their mechanism of action, yet this terminology is often inconsistently defined and applied (Sachdev et al., 2021). Here, we make a distinction between bivalent compounds, in which both constituent pharmacophores target GPCR orthosteric sites and bitopic compounds, in which one (or more) pharmacophore targets a nonorthosteric site. We further subdivide this collection of compounds into: *cis*-bitopic ligands, which selectively target the primary orthosteric site and a secondary (or allosteric) site within a single receptor molecule (Figure 1E), and *trans*-bitopic ligands, which target the orthosteric site in one receptor molecule and a secondary site in a separate receptor molecule (Figure 1F). Such binding scenarios offer mechanisms in which a bitopic ligand first binds to one protomer, thereby increasing local concentration at the second co-localized receptor, which promotes binding of the tethered pharmacophore to its target. The simple co-expression of two receptors on a single cell can therefore be exploited in applying bitopic ligands to harness the thermodynamic and kinetic advantages of the bivalent binding relative to monovalent binding. These benefits can be leveraged to realize high affinity and selective engagement of receptor pairs over either monomer alone.

### 2.2 | Applications and lessons from small-molecule multivalent ligands for GPCRs

There is an extensive literature describing the unique and desirable properties of small molecule bivalent and bitopic ligands that target GPCRs (Lane et al., 2013; Newman et al., 2020; Valant et al., 2012). *Cis*-bitopic ligands can be effectively deployed when a secondary (allosteric) binding site is accessible and spatially proximal to the orthosteric site. This scenario is often realized when a secondary site is located on the extracellular surface of the receptor and is structurally characterized. One prominent collection of targets that meet these criteria is the muscarinic acetylcholine receptor family (Jakubik & El-Fakahany, 2020). There are several examples of compounds that engage muscarinic receptor allosteric sites, one of which is found near the extracellular face of the receptor. The availability of well-characterized compounds that engage allosteric binding sites is highly beneficial for bitopic ligand design. For example, a bitopic ligand for the muscarinic M_2_ receptor (THRX-160209) demonstrated a substantial gain in affinity compared with its constituent orthosteric and allosteric building blocks (Steinfeld et al., 2007). A different bitopic ligand comprised of an orthosteric ligand for muscarinic receptors (iperoxo) linked to a positive allosteric modulator demonstrated unexpected selectivity for certain receptor subtypes (M_2_/M_4_) (Wakeham et al., 2022). Muscarinic receptors serve as a prominent testing ground for the development of bitopic ligands as the highly conserved orthosteric acetylcholine-binding pocket has limited the development of receptor subtype-selective ligands, thus demanding novel approaches. The above examples demonstrate that bitopic ligands that engage an allosteric receptor site can exhibit improved subtype selectivity and/or enhance binding affinities.

Dual engagement of two binding sites by bitopic ligands is desirable, but can be challenging to realize, with linker design playing a prominent role. Studies of the adenosine A_1_ receptor revealed that the binding of a bitopic ligand was sensitive to competition from a monomeric allosteric pharmacophore counterpart (Narlawar et al., 2010). This observation suggested that the bitopic ligand may not concomitantly occupy both sites, but rather toggle between the two binding sites, allowing an opportunity for monovalent engagement to outcompete bitopic binding and cause rapid dissociation. This property was attributed to the use of a linker of insufficient length between the two pharmacophores. The importance of linkers is further illustrated by studies targeting 5-HT GPCR assemblies (Choi et al., 2008; Lezoualc’h et al., 2009; Soto et al., 2018). In one such study, the potency of bivalent antagonist conjugates varied as a function of linker length, with intermediate linkers conferring the highest activity (Soto et al., 2018). In another study the stereochemistry and composition of linkers in bitopic ligands affected their activity at dopamine receptors (D_2_ and D_3_ receptors) (Semeano et al., 2024). Linker rigidity or flexibility can also dictate the conformational propensities of bitopic ligands, and consequently, their ability to engage the intended binding sites. For example, rigid oligoproline-based linkers have been used to impose geometric constraints on bitopic ligands to promote dual engagement of two gastrin-releasing peptide receptors (GRP-R; now known as bombesin BB_2_ receptors) protomers, leading to an altered signalling profile (Romantini et al., 2021). Bitopic ligands in which the geometric constraints for simultaneous engagement of multiple binding sites are met, as exemplified by bitopic ligands for μ-opioid receptors (Faouzi et al., 2023), can demonstrate potent and pathway-selective signalling (biased agonism).

Ligands engaging targets through a *trans*-bitopic mechanism are valuable tools for preferentially engaging pairs of co-expressed receptors. This approach is exemplified by ligands targeting the cannabinoid CB_1_ receptor–μ-opioid receptor pair (Le Naour et al., 2013). CB_1_ and μ receptors are co-localized in specific regions of the CNS and are involved in nociceptive signalling. Bitopic ligands comprised of a selective μ receptor agonist linked to a CB_1_ receptor-selective antagonist/inverse agonist via spacers of varied lengths were studied. Immunofluorescence microscopy provided evidence that such bitopic ligands can bridge CB_1_ receptor and μ receptor protomers on the surface of a single cell. A similar strategy was applied to co-target dopamine D_3_ receptors and neurotensin NTS_1_ receptors (Budzinski et al., 2021). In this example, an NTS_1_ receptor agonist was linked to a dopamine-mimetic pharmacophore. Bitopic ligands displayed a marked preference for binding D_3_–NTS_1_ receptor heteromers over D_2_–NTS_1_ receptor heteromers. Bitopic engagement of D_3_–NTS_1_ receptors also promoted internalization of the D_3_ receptor which did not occur with monovalent ligands. These observations demonstrate the capacity of bitopic ligand to confer useful and interesting pharmacological properties, such as receptor subtype selectivity within closely related receptor families, and emergent changes in receptor trafficking properties. These examples, and others, have provided valuable insights into the importance of spatial constraints, avidity effects and binding site requirements for realizing useful properties in targeting receptors such as GPCRs. Such principles can be applied efforts to design multivalent binders that incorporate biologically-derived binding elements, such as those discussed below.

## 3 | EXPANDING TARGET RANGE WITH BIOLOGIC-BASED BITOPIC CONJUGATES

### 3.1 | Antibody-based scaffolds for GPCR targeting

Despite substantial progress in developing small molecule bitopic ligands for GPCRs, such efforts remain a significant challenge, in large part because most GPCRs have poorly characterized secondary binding sites. The use of biological affinity reagents (biologics) that can bind protein surfaces not easily amenable to small molecule engagement offers new avenues for the design of multivalent and bitopic receptor ligands (McGonigle, 2025). The modularity, high specificity and ability to engage unique extracellular epitopes marks biologics as versatile scaffolds for targeting GPCRs (Peterson et al., 2023). In the sections that follow, we discuss principles and design strategies for application of biologics, with specific reference to case studies involving bitopic GPCR targeting.

**FIGURE 2:**
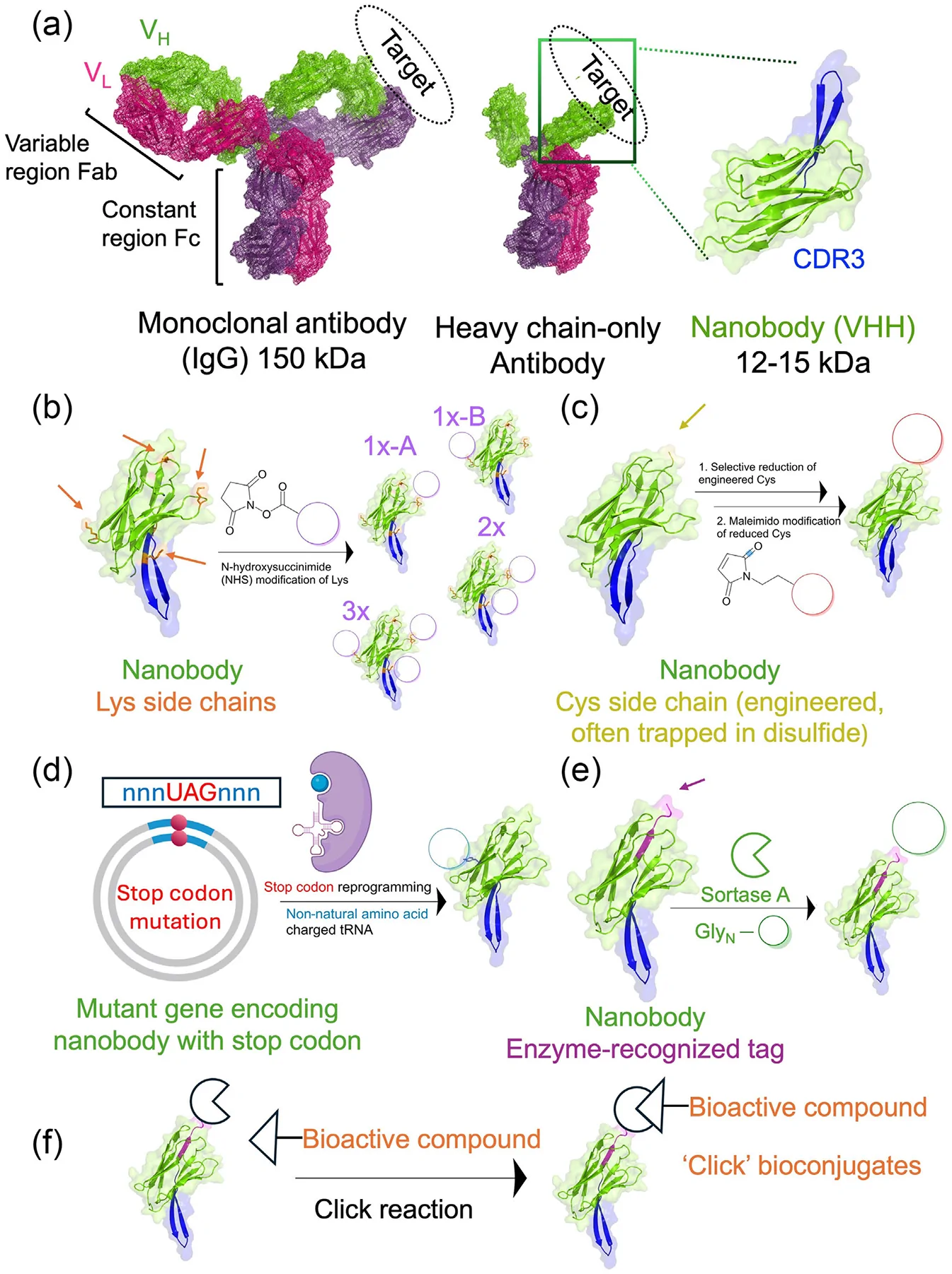
Antibody architecture and modification via conjugation chemistries. (A) Structural comparison of a full-length monoclonal antibody (Ab), a heavy-chain-only camelid Ab, and a single-domain antibody (nanobody, Nb) fragment. (B–E) Conjugation methods applied to generate bitopic conjugates with Nb building blocks. Methods include (B) stochastic modification of lysine side chains using N-hydroxysuccinimide chemistry, (C) incorporation of engineered cysteine residues for labelling with functionalized maleimides, (D) stop-codon suppression via genetic code reprogramming to site-specifically incorporate non-natural amino acids, (E) enzymatic labelling for site-specific modification, exemplified by sortase A-mediated ligation and (F) ‘click’ chemistries for modifying proteins labelled with biorthogonal handles. Structural templates used for schematic generation were obtained from published structures (Ab, PDB 5DK3 and Nb, PDB 8QOT).

Antibodies represent the most broadly applied and extensively developed class of biologics (Figure 2A) (Qian et al., 2023). Biologics are often characterized by exceptional affinity and specificity toward surface-exposed epitopes in membrane embedded proteins. Such epitopes can be difficult to target with small molecules. Antibodies offer additional compelling pharmacological properties, including prolonged circulatory half-lives and favourable pharmacokinetics that support sustained therapeutic activity. The successful application of antibodies in oncology, immunology and infectious disease underscores both their versatility and therapeutic utility (Carter & Rajpal, 2022). Some successes have been recorded in deploying antibodies to bind and neutralize GPCR ligands, such as calcitonin gene-related peptide for the treatment of migraine (Versijpt et al., 2025). In a few examples, antibodies that directly bind GPCRs have entered clinical evaluation or have been approved for therapeutic application; however, most of these antibodies do not modulate receptor function directly, but rather function in multispecific constructs, as described in subsequent sections (Hutchings et al., 2026; Peterson et al., 2023). Despite these successes, the use of antibodies in biological processes controlled by GPCR function has remained limited. The use of antibodies to alter GPCR function is challenging, largely because these receptors often present only limited extracellular surface for engagement, with most of the protein embedded in the plasma membrane. For example, class A GPCRs, the largest and most diverse GPCR subfamily that includes the highly studied chemokine receptors, typically only display short fragments as potential extracellular epitopes. Notable exceptions include class B (secretin family) and Class C GPCRs, which possess large extracellular domains that provide greater accessibility for antibody binding (Yang et al., 2021). Complicating matters further, many GPCR orthosteric sites are located in parts of the receptor that are embedded in the plasma membrane, although prominent exceptions such as class C GPCRs and two of the relaxin receptors (RXFP1/2) have been documented. As antibodies engage their target through relatively dispersed binding surfaces (paratope), their ability to access orthosteric (or other poorly accessible) GPCR epitopes is often restricted (Schlimgen & Volkman, 2025). Technologies to enable the development of functional antibodies capable of modulating GPCR signalling would open new opportunities. Achieving this, however, remains a formidable challenge.

Limitations with conventional antibodies have spurred interest in alternative scaffolds. Other targeting scaffolds such as DARPins (Milovnik et al., 2009), nucleic acid-based aptamers (Takahashi, 2022) and cyclic peptides (Li et al., 2025) have been investigated for GPCR recognition, although each of these targeting moieties have been applied in relatively few examples. Variable fragments of heavy-chain-only antibodies (VHHs) or nanobodies, which are single-domain antibody fragments derived from the variable domain of heavy-chain-only camelid (camels, alpacas and llamas) antibodies, have been studied more extensively (Figure 2A) (Cheloha, Harmand, et al., 2020; Sachdev, Roy, Saha, et al., 2025). Nanobodies are substantially smaller than antibodies (about 15 vs. 150 kDa), retain the high affinity and specificity of antibodies, and possess characteristics advantageous for targeting GPCRs (Schlimgen & Volkman, 2025). The interaction of nanobodies with their targets occurs via three complementarity-determining regions (CDRs) compared with six in conventional antibodies. The extended length and structural diversity of the nanobody CDR3 can compensate for the reduced number of loops to offer affinity and specificity comparable to traditional antibodies (Figure 2A). Importantly, the CDR architecture of nanobodies often produces a convex paratope surface, which can reach concave or buried epitopes, including orthosteric ligand-binding pockets or other pockets within GPCRs that are inaccessible to conventional antibodies (Schlimgen & Volkman, 2025). This feature is manifest in the frequent use of nanobodies to specifically stabilize receptor conformational states (active or inactive), allowing their application in methods to monitor the dynamics and localization of GPCR activation (Bock et al., 2025) or structural biology studies (Salom et al., 2024). Another strength of nanobodies is their ability to form extensive intermolecular contacts that extend beyond the classical ligand-binding pocket. Empirical observations show that nanobodies that bind to GPCRs outside the orthosteric site tend to engage the receptor extracellular face through the interaction of CDR1 and CDR2 loops with the receptor N-terminus and extracellular loops (Salom et al., 2024; Wingler & Feld, 2022).

Unlike conventional antibodies, which are often costly to produce, requiring production in specialized cell types or isolation from animal sera, nanobodies are encoded by a single open reading frame, correspond to a single protein domain, and can be expressed efficiently in prokaryotic and eukaryotic hosts, including bacteria and yeast (Cheloha, Harmand, et al., 2020). This ease of recombinant production enables modular construction approaches in which nanobodies can be systematically linked to pharmacological probes, peptides or chemical warheads using either genetic fusions (Yan et al., 2025) or chemical labelling methods (Schumacher et al., 2018). Neither heavy-light-chain pairing nor glycosylation are required for nanobody function, which confers additional flexibility for nanobody engineering. As an example, a fusion between a GLP-1 receptor agonist peptide and two nanobodies, one targeting the receptor and the other targeting serum albumin to extend circulatory half-life, was readily produced by recombinant expression and effectively activated GLP-1 receptors *in vitro* and in mice (Pan et al., 2020). Such modularity also opens the door to generating collections of bitopic or multivalent nanobody conjugates (Wang et al., 2022) tailored for GPCR targeting. Combining nanobody modularity with the expanding repertoire of bio-orthogonal chemistries (discussed below) offers a path toward precise construction of multivalent conjugates with useful properties. Collectively, these properties establish nanobodies as a versatile platform for addressing GPCR pharmacology.

### 3.2 | Conjugation chemistry: combining biologics and bioactive synthetic molecules

Although some GPCR-targeting antibodies (and nanobodies) have been reported to modulate receptor function, most commonly as antagonists, many such binders do not directly modulate signaling (Salom et al., 2024; Schlimgen & Volkman, 2025). Robust methods that facilitate linkage of antibodies with bioactive compounds are highly desirable, as this methodology provides a modular strategy to convert receptor-binding antibodies into ligands with tunable signalling activity (Walsh et al., 2021). Bioconjugation refers to the process of appending one biomolecule (e.g., an antibody or nanobody) to another functional entity to generate a hybrid conjugate. The successful design of biologic bitopic conjugates requires careful consideration of several variables, including the choice of the antibody/nanobody binder, the composition and characteristics of the ligand moiety (agonist, antagonist, full or partial agonist), the length and chemical nature of the linker and the sites at which the linker is attached. Design principles established in the generation of bioactive bitopic small molecule ligands (see above) are useful in guiding biologic conjugate design. Each of these variables can have profound effects on bioconjugate properties including signalling potency, receptor selectivity, therapeutic efficacy and other pharmacological parameters.

#### 3.2.1 | Genetic fusion of biologics with bioactive ligands

A rich body of literature exists describing antibody conjugation strategies, most notably in the development of antibody–drug conjugates (ADCs). ADCs are typically used for the delivery of cytotoxic small molecules to cancer cells (Walsh et al., 2021). We will not review this literature in depth here but instead focus on examples in which GPCRs are targeted or specialized bioconjugation chemistries are deployed. Genetic fusion offers a conceptually straightforward strategy for creating biologic conjugates targeting cell surface receptors (Silver et al., 2021; Zheng et al., 2022). In this approach, bioactive polypeptides are fused to antibodies or antibody fragments through production of a synthetic DNA encoding a fusion protein and subsequent recombinant expression. Such approaches are only applicable in cases in which the GPCR being targeted can be activated by a genetically encodable peptide (or protein). While conceptually straightforward, such fusions often suffer from reduced stability or folding efficiency, requiring extensive empirical optimization. For example, the successful generation of a bitopic fusion between an IgG that targets the soluble protein PCSK9 and a polypeptide agonist for GLP-1 receptors demanded careful engineering to achieve a stable, functional product (Chodorge et al., 2018). Because purely genetic approaches are usually restricted to the naturally encoded amino acid repertoire, they cannot easily incorporate chemical modifications such as C-terminal amidation, unusual cyclization chemistries or non-natural residues that confer proteolytic stability, which are essential for robust bioactivity in many cases. As a result, generating genetically encoded fusions containing bioactive peptides that depend on such modifications remains challenging.

#### 3.2.2 | Chemical and site-specific conjugation strategies

Limitations associated with genetic fusions have motivated broader exploration of conjugation strategies that allow the incorporation of chemically synthesized components. Such conjugates are often produced by linking synthetic bioactive compounds to electrophilic moieties that can react with the side chains on antibodies to form a covalent linkage (Taylor et al., 2022; Walsh et al., 2021). Conventional antibodies contain numerous residues with nucleophilic side chains, such as lysine and cysteine, that can be exploited for chemical modification (Figure 2B). Early antibody conjugation strategies exploited these reactive residues through coupling chemistries that include *N*-hydroxysuccinimide ester-mediated acylation of lysine (Figure 2B) and maleimide-mediated labelling of reduced cysteine thiol side chains (Figure 2C). While this approach is typically effective in providing antibody-based bioconjugates, such stochastic approaches yield heterogeneous product mixtures with uneven distribution of synthetic payloads. Such heterogeneity complicates pharmacological characterization, can cause aggregation and precipitation of conjugates and can compromise performance *in vivo*. An illustrative example is seen in studies of a nanobody (HN3) targeting the tumour-associated antigen glypican-3, a target overexpressed in liver cancer (Fayn et al., 2023, 2025). Stochastic labelling of HN3 with a radiolabel led to non-specific accumulation *in vivo*, whereas site-specific labelling enabled selective payload delivery with improved tumour uptake and biodistribution. The stochastic labelling of antibodies (or nanobodies) with high molecular weight compounds (such as peptidic GPCR ligands) is likely to be particularly problematic, leading to highly heterogeneous mixtures and risking conjugate aggregation.

The shortcomings of stochastic labelling approaches motivated the development of site-specific conjugation chemistries for bioconjugate production (Taylor et al., 2022; Walsh et al., 2021). One widely used approach involves the introduction of an unnatural cysteine residue that does not engage in structural disulfide bond formation and is thus available for labelling. Many proteins, including antibodies and nanobodies, do not natively possess an unpaired Cys residue, thus enabling site-specific labelling using a naturally occurring amino acid. Common functional groups for cysteine modification include maleimide and α-halo carbonyl derivatives, which form thioether linkages with cysteines and are widely commercially available (Figure 2C). Another approach for site-specific labelling relies on the incorporation of unnatural amino acids with reactivity profiles that are orthogonal to naturally occurring amino acids (Pigula & Schultz, 2024). DNA stop-codon suppression methods allow for the site-specific incorporation of amino acids such as *p*-azido-phenylalanine or *p*-acetyl-phenylalanine into proteins (Figure 2D). For example, a mutant of the monoclonal antibody trastuzumab (Herceptin, Her2 binder) incorporating *p*-acetyl-L-phenylalanine at specific positions in the constant region enabled site-specific conjugation through labelling with an alkoxy-amine conjugated auristatin derivative to provide an imine-linked antibody–drug conjugate (Axup et al., 2012). Such approaches highlight the utility of non-natural amino acid incorporation via translational reprogramming; however, challenges remain. Experimental protocols can be technically demanding, the modified residues may compromise protein folding or stability, and not all sites of mutation can be accommodated with retention of target binding.

Protein modification using enzymatic chemistry offers an alternative for site-specific labelling (Debon et al., 2023). Such approaches typically rely on appendage to peptide tags onto proteins of interest via genetic fusion. These tags are selectively recognized and modified by enzymes to site-specifically append bioactive compounds or chemical tags that can be used for further synthetic elaboration (Figure 2E). Several enzymes have been employed for site-specific labelling approaches including formylglycine-generating enzyme (Boschanski et al., 2021), transglutaminase (Deweid et al., 2019), sortase A (Amacher & Antos, 2024) and others (Falck & Müller, 2018). Here, we focus on Sortase A (SrtA), as its conjugation chemistry is widely applied in the construction of bitopic conjugates applied in targeting GPCRs (Braga Emidio & Cheloha, 2024b). SrtA recognizes the LPXTG peptide motif (X being any amino acid besides Pro) and cleaves between the threonine and glycine residues to generate a transient thioester intermediate. This intermediate is then resolved by nucleophilic attack from an oligoglycine-functionalized partner, forming a new amide bond linkage in the peptide backbone at the protein C-terminus. The straightforward implementation of sortagging has enabled installation of diverse ligands, probes or bio-orthogonal chemical handles onto antibodies or nanobodies, without perturbing structure or binding.

#### 3.2.3 | Bio-orthogonal and chemoselective chemistry

Many of the labelling technologies described above are not used to directly append biologically active ligands to antibodies or nanobodies, but instead for the introduction of moieties that can be used in subsequent chemoselective reactions for further synthetic elaboration. Bio-orthogonal chemistry, which refers to reactions between two moieties that are mutually reactive yet remain inert to functionalities naturally found in biological contexts, has emerged as a highly effective toolbox of reactions for efficiently linking two (or more) biomolecules (Scinto et al., 2021). Prominent examples include copper-catalysed azide–alkyne cycloaddition (CuAAC) and a metal-free variant (strain-promoted azide–alkyne cycloaddition, SPAAC), collectively known as ‘click’ chemistry (Pickens et al., 2018) (Figure 2F). These reactions are highly selective, rapid and proceed efficiently in mild aqueous conditions, making them particularly attractive for bioconjugation. Additional chemoselective reactions have been developed and applied for bioconjugation, including tetrazine–*trans*-cyclooctene cycloadditions (Tomarchio et al., 2024), oxime and hydrazone formation (Kölmel & Kool, 2017), Staudinger ligation (Bednarek et al., 2020) and sulphur–fluoride exchange (SuFEx) chemistry (Hansen et al., 2025). Such reactions can also be applied in situ to generate bioconjugates on demand, in the biological context of interest or at their site of action (Saha & Cheloha, 2024). Below we describe case studies in the application of chemoselective chemistries in the generation of bitopic ligands for targeting GPCRs, with a comprehensive list provided in Table 1.

## 4 | CASE STUDIES IN BIOLOGICS-BASED BITOPIC GPCR TARGETING

### 4.1 | Peptide–biologic conjugates

#### 4.1.1 | Parathyroid hormone receptor 1

**FIGURE 3:**
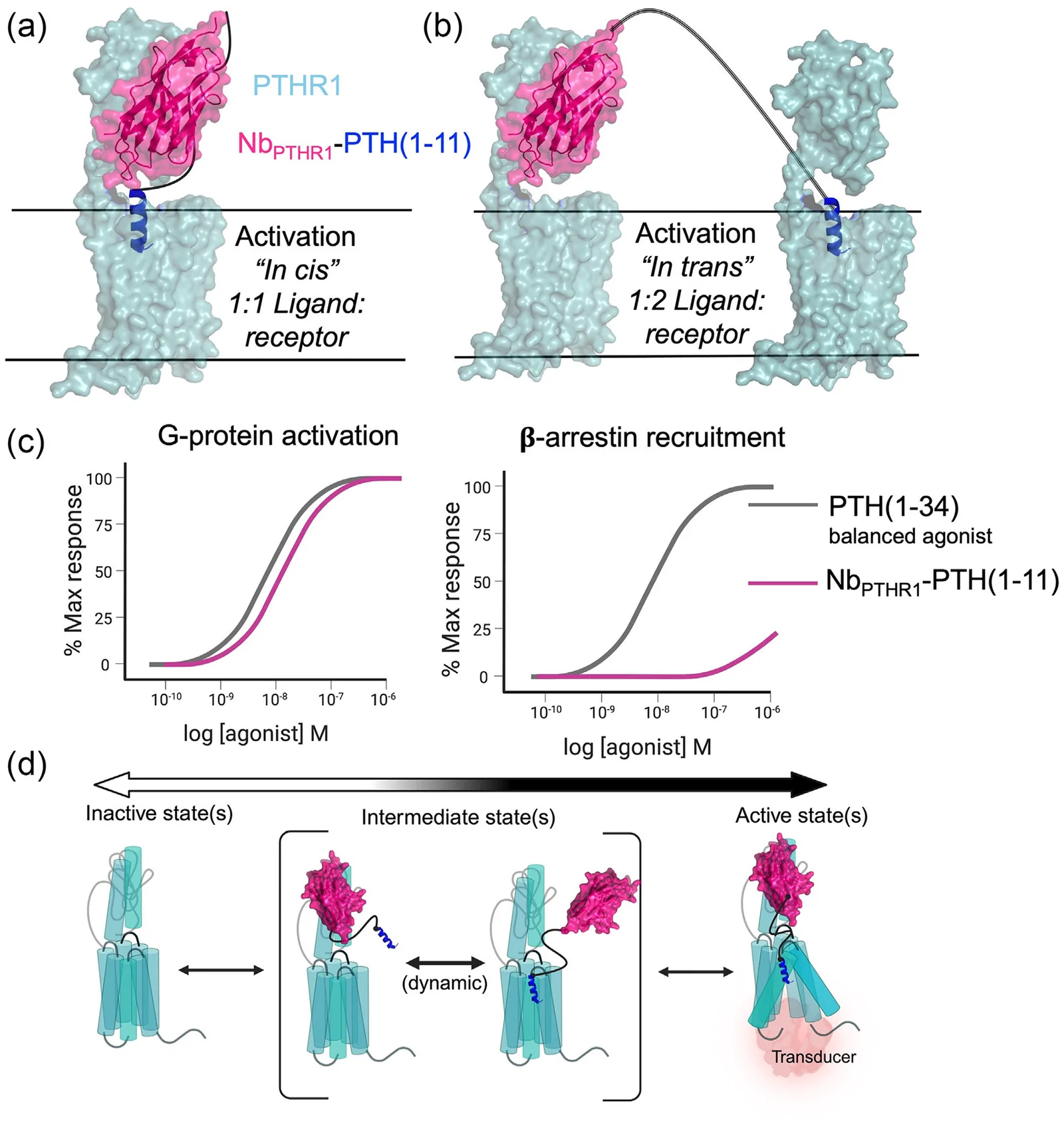
Nanobody–ligand bitopic conjugates for PTH1 receptors. (A) Schematic of a Nb–PTH ligand conjugate (Nb in pink, PTH in blue) engaging PTH1 receptors (cyan) with a 1:1 ligand-to-receptor ratio (‘activation *in cis*’). (B) Alternative mechanism of receptor engagement in which the Nb–PTH conjugate bridges two receptor protomers through a 1:2 ligand-to-receptor ratio (‘activation *in trans*’). (C) Schematic representation of the pharmacological profiles of a Nb–PTH conjugate and a conventional PTH(1–34) agonist. Note differences in cAMP and β-arrestin responses. (D) Schematic providing a hypothetical model of the dynamic interaction of a nanobody–ligand conjugate with PTH1 receptors. A structural template for PTH1 receptors was obtained from experimental data (PDB 6FJ3). Models of the Nb–PTHR1 complexes were generated using AlphaFold 3 (Abramson et al., 2024).

The parathyroid hormone receptor 1 (PTH1 receptor) is a prototypical class B GPCR that engages its ligand through a two-site binding mechanism (Cheloha et al., 2015). The PTHR1 extracellular domain (ECD) mediates high affinity ligand binding without direct involvement in the induction of signalling. In contrast, interactions between the ligand and transmembrane domain (TMD) occur with lower affinity but are essential for signal transduction (Cheng et al., 2023). The large size of the PTHR1 ECD, which is unusual among the GPCR superfamily, constitutes an appealing site for antibody or nanobody docking in the context of multivalent conjugates. Animal immunization with purified and refolded PTH1 receptors resulted in the generation of nanobodies that recognize epitopes in the ECD (Adams et al., 2016), which are distinct from the TMD-embedded orthosteric ligand-binding site (Figure 3A). As such, these nanobodies function predominantly as neutral binders, meaning they do not strongly activate or inhibit the receptor. Traditionally, such pharmacologically silent binders have been suboptimal for the development of GPCR-modulating agents; however, they constitute valuable building blocks for the development of multivalent conjugates as described below.

Work from our group has tested whether linking PTH1 receptor ligands to nanobodies that bind their targets without intrinsic signalling activity could manifest enhanced or unusual pharmacological properties (Cheloha, Fischer, et al., 2020; Sachdev et al., 2024). Two nanobodies (NbPTHR1 and NbPTHR1X2), which bind to sites on the ECD of PTH1 receptors that do not overlap, lack either pronounced agonist or antagonist activity. We asked whether linking these nanobodies to peptide ligands (PTH1–11 or PTH1–14), which display modest agonist potency and low specificity between two PTH-receptor subtypes (PTH1/PTH2), would result in improved agonist properties. The nanobodies were site-specifically linked to cargoes using a combination of enzymatic labelling (sortagging) and click chemistry to produce Nb-PTH(1–11) or Nb-PTH(1–14) conjugates (Table 1). These conjugates exhibited potencies that were enhanced by over 1000-fold relative to unconjugated ligand counterparts. Similar results were achieved using full-size monoclonal antibodies in place of the nanobodies (Cheloha et al., 2021). One optimized conjugate (NbPTHR1–PTH[1–14]) exhibited picomolar level potency at PTHR1 and was inactive at PTH2 receptors, whereas PTH(1–34) and PTH(1–14) were much less selective. Furthermore, NbPTHR1–PTH(1–14) showed activity in a mouse-based assessment of PTH1 receptor activation (Cheloha, Fischer, et al., 2020).

Mechanistic studies were conducted to understand how Nb-PTH conjugates signal (Sachdev et al., 2024). Two engineered variants of PTH1 receptors that require dual engagement of both versions of receptor protomer for potent signalling were co-expressed in a population of cells. In this context, the Nb–PTH(1–11) conjugate stimulated robust cAMP responses whereas PTH(1–34) did not. These results suggest that, unlike conventional PTH1 receptor ligands that engage a single receptor protomer, nanobody–ligand conjugates may engage multiple, co-expressed receptor protomers (Figure 3B). This feature of nanobody–ligand conjugates offers wide-ranging prospects for selective targeting co-expressed GPCR pairs (see below).

We also measured the pharmacological activity of nanobody–ligand conjugates in stimulating several signalling pathways downstream of PTH1 receptor activation (Sachdev et al., 2024). Canonical PTH1 receptor agonists engage Gα_s_-, Gα_q_- and β-arrestin–mediated pathways, with evidence of signalling from endosomal compartments. The Nb–PTH(1–11) conjugates induced robust signalling through the Gα_s_-pathway, as indicated by cAMP production (Figure 3B). These cAMP responses persisted even after a ligand washout phase, suggesting slow dissociation kinetics for Nb-PTH(1–11) conjugates. The same conjugates showed substantially diminished capacity to induce β-arrestin recruitment, activate Gα_q_ signalling and induce endosomal signalling relative to the PTH(1–34) prototype agonist (Figure 3C). Neither nanobody alone nor Nb-PTH(1–11) conjugates induced receptor internalization. The mechanism of biased agonism for Nb-PTH(1–11) conjugates is unknown. One possibility is that the ‘activation *in trans*’ mechanism described above (Figure 3B) directly leads to pathway-selective signalling. Another non-exclusive possibility is that bivalent engagement of a single receptor molecule results in a dynamic mode of interaction (Figure 3D). The formation of a rapidly interchanging ensemble of intermediate- and fully active receptor states could also lead to pathway-selective signalling (Figure 3D). These studies established selected Nb-PTH(1–11) conjugates as the most highly pathway-selective (or biased) agonists of PTH1 receptor described to date. Such biased agonists offer exciting prospects for engagement of signalling pathways related to therapeutically beneficial responses, while avoiding on-target, off-pathway side effects.

#### 4.1.2 | Glucagon-like peptide 1 receptor and glucose-dependent insulinotropic polypeptide receptor

**FIGURE 4:**
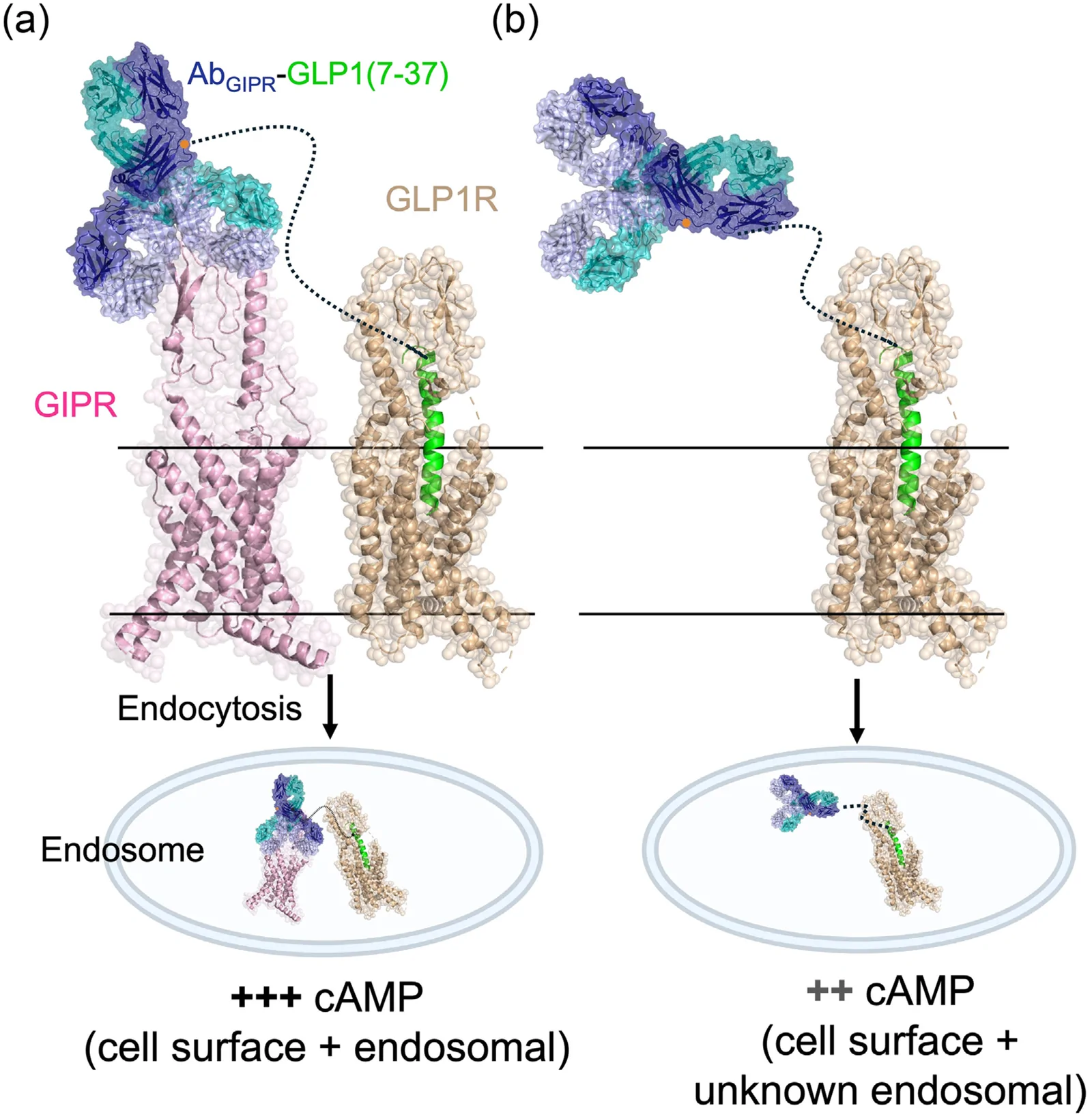
Dual targeting of GLP-1 receptors and GIP receptor by an Ab_GIPR_–GLP1 conjugate. (A) Schematic of a bitopic conjugate simultaneously engaging GIP receptors (GIPR) and GLP-1 receptors (GLP1R), with the antagonist antibody (blue) binding GIP receptors (light pink) and the GLP-1 ligand (green) engaging GLP-1 receptors (wheat). Dual engagement promotes cAMP production at the plasma membrane and endosomes, resulting in greater overall signalling output. (B) Scenario in which the ligand engages GLP-1 receptors in the absence of an Ab_GIPR_ interaction, producing a lower level of cAMP production, predominantly at the cell surface. Structural templates used for schematic generation were obtained from experimental data (GLP-1 receptor, PDB 6VCB and GIP receptor, PDB 7FIN).

The GLP-1 receptor and the GIP receptor are class B GPCRs stimulated by their respective incretin hormones, GLP-1 and GIP. These receptors have garnered increasing attention as targets in the treatment of Type-2 diabetes and obesity (Campbell et al., 2023). Interestingly, recent studies show that agents that exhibit either dual GLP-1/GIP receptor agonism or GLP-1 receptor agonism/GIP receptor antagonism can robustly induce weight loss in rodents (Gutgesell et al., 2025; Liu et al., 2025). While GLP-R/GIPR co-agonism is typically engaged through the action of a single polypeptide, GLP-1 receptor agonism/GIP receptor antagonism activity was realized using antibody-based bispecific conjugates (Lu et al., 2021; Véniant et al., 2024). In this approach, a GIP receptor antagonist antibody was mutated to incorporate a free cysteine. A chemically synthesized GLP-1 analogue containing a non-natural amino acid (Aib, to enhance stability) was functionalized with an electrophilic group (bromoacetyl) to enable linkage with the antibody through a Cyselectrophile reaction (Véniant et al., 2024). As expected, the conjugate (Ab_GIPR_-GLP1, also known as AMG133, Table 1) exhibited antagonistic activity in GIP receptor-expressing cells and agonistic activity in GLP-1 receptor-expressing cells (Lu et al., 2021) (Figure 4A). A control conjugate, in which the peptide was linked to an antibody that does not bind GIP receptors, was evaluated in these same assays. The control antibody-GLP1 analogue conjugate was less active than the same GLP-1 analogue peptide alone. This observation is consistent with prior findings from our group, which showed that linking an GPCR ligand to a non-binding nanobody greatly blunted agonist properties, likely to be due to steric interference imposed by the linked antibody or nanobody (Cheloha, Fischer, et al., 2020; Sachdev et al., 2024).

Ab_GIPR_–GLP-1 conjugates were also evaluated in cells co-expressing GLP-1 and GIP receptors (Lu et al., 2021) (Figure 4A). When both receptors were expressed, the conjugate exhibited greater potency for cAMP production than GLP-1 alone. When only GLP-1 receptors were expressed, signalling for Ab_GIPR_–GLP-1 was still observed but at reduced levels, underscoring the role of high affinity antibody binding in potent agonist responses (Figure 4B). Assessment of receptor trafficking upon dual GLP-1/GIP receptor engagement by Ab_GIPR_–GLP-1 showed β-arrestin recruitment and internalization of both receptors (Lu et al., 2021). Blockade of receptor internalization attenuated cAMP production by Ab_GIPR_–GLP-1, suggesting an important role for endocytic signalling in conjugate bioactivity. However, in cells expressing only GLP-1 receptors, the role of internalization and endosomal signalling for responses observed upon treatment with Ab_GIPR_–GLP-1 remain unclear.

The *in vivo* efficacy of the Ab_GIPR_–GLP-1 conjugate was assessed in mice and non-human primates (Lu et al., 2021; Véniant et al., 2024). Administration of the conjugate resulted in significant reduction in body weight compared to either the GIPR-targeting antibody or peptide ligand alone, suggesting a synergistic effect from dual receptor engagement. Although the precise mechanism underlying this enhanced efficacy remains unclear, biodistribution studies using DOTA-labelled Ab_GIPR_–GLP-1 conjugate revealed increased accumulation of the conjugate in organs including the pancreas, liver, lung and bone marrow (Lu et al., 2021). These findings suggest that tissue-specific receptor engagement by the conjugates may contribute to their superior performance.

Separate work from our group (Sachdev et al., 2024) and others (Liu et al., 2021) describe alternative bitopic conjugates targeting GLP-1 receptors. A GLP-1 receptor-specific nanobody was site-specifically linked to an analogue of the GLP-1 peptide that contained mutations that attenuated receptor affinity, in an approach analogous to that used to produce the PTH1 receptor-targeting conjugate described above. This NbGLP1R-GLP1_mut_ conjugate (Table 1) differs from Ab_GIPR_–GLP-1 (Véniant et al., 2024) construct both in the structure of the GLP1 peptide used and in target specificity (dual GLP-1 receptor binding vs. combination GLP-1 receptor/GIP receptor binding). Pharmacological characterization of the NbGLP1R–GLP-1_mut_ conjugate revealed potent stimulation of Gα_s_-cAMP signalling with negligible β-arrestin recruitment. In another example, the genetic fusion of GLP-1(7–36) to the N-terminus of a GLP-1 receptor-specific antibody provided a conjugate (TB59–2, Table 1) that exhibited strong Gα_s_-cAMP signalling at GLP-1 receptors and activity *in vivo* (Liu et al., 2021). In contrast, TB59–2 exhibited reduced activity in stimulating the recruitment of β-arrestin to GLP-1 receptors, relative to GLP-1 alone, a trend analogous to that observed with Nb_GLP1R_–GLP-1_mut_ (Sachdev et al., 2024). The factors that dictate whether bitopic conjugates stimulate arrestin recruitment, or the internalization of receptor complexes, remain to be delineated. In total, these examples demonstrate that biologic–peptide conjugates can expand the pharmacological properties accessible to incretin receptor ligands beyond those achievable with peptide agonists alone. Such conjugates offer the prospect of elucidating how receptor engagement, tissue-selective targeting and signalling bias contribute to therapeutic efficacy and to the development of the next-generation of medicines.

#### 4.1.3 | Neurokinin-1 receptor

The neurokinin NK_1_ receptor is a class A GPCR from the tachykinin receptor family (Johnson et al., 2017). These receptors are endogenously targeted by peptide agonists to regulate nociception and inflammation. Unlike class B GPCRs, which possess a well-defined ECD that can serve as a distinct anchoring site for nanobody or antibody engagement, class A receptors such as the NK_1_ receptors typically lack this architectural feature, presenting a challenge in the identification of target-specific binders. As no nanobody binder has yet been identified for this receptor, our group produced a receptor variant in which epitope tags were genetically appended at N-terminus (extracellular portion) of NK_1_ receptors (Braga Emidio & Cheloha, 2024a). Three orthogonal epitope tags (6E, Alfa and BC2) bound by distinct nanobodies were appended in tandem to produce a triply epitope tagged receptor.

Nanobodies targeting each of these epitope tags were conjugated to NK_1_ receptor peptide agonists (NKA or SP_6-11_) via sortase-mediated ligation (Table 1) (Braga Emidio & Cheloha, 2024a). These conjugates were tested for their ability to stimulate signalling pathways downstream of NK_1_ receptor activation, including β-arrestinrecruitment and Gα_s_- or Gα_q_-mediated responses. The panel of nanobody–ligand conjugates exhibited varying activity dependent on the identity of the nanobody and ligand used for conjugate synthesis, as well as the pathway under study. Most nanobody–ligand conjugates showed increased potency but reduced maximal effect in the Gα_s_-induced cAMP production, compared to their parent ligands. In contrast, most conjugates exhibited full efficacy with variable potency for Gα_q_- signalling and β-arrestin-recruitment. Further analysis revealed that, despite lower maximal activity at Gα_s_-pathways, several conjugates demonstrated more enduring cAMP signalling than the parent peptide, which also translated into stronger transcriptional outputs. The origin of variation among the different nanobody–ligand conjugates is unclear but may relate to variation in the positioning (proximal vs. distal) of the nanobody epitope tag at the receptor N-terminus. These findings highlight the complexity of nanobody- (or antibody-) ligand conjugate signalling activity and the requirement for empirical evaluation of both receptor activity and dynamics. Future studies will benefit from new techniques to identify nanobody binders that recognize the wild-type NK_1_ receptors and other target receptors of interest.

#### 4.1.4 | Angiotensin II AT_1_ receptors

The signalling of bitopic nanobody–ligand conjugates at AT_1_ receptors was assessed by using an engineered receptor with an N-terminal epitope tag (6E) appended to the receptor via genetic fusion. Although nanobodies targeting AT_1_ receptors have been characterized (McMahon et al., 2020; Skiba et al., 2024, 2025), they act as antagonists thereby complicating efforts to generate nanobody–ligand conjugates with agonist activities. A panel of bitopic ligands was generated by site-specifically conjugating Nb_6E_ to a series of structurally similar but functionally diverse peptide ligands (Ang II, TRV027, TRV055 and TRV056) for AT_1_ receptors (Braga Emidio et al., 2023) (Table 1). These conjugates were evaluated for signalling via Gα_q_ and in β-arrestin recruitment assays. While all bitopic ligands exhibited enhanced potency relative to their parental counterpart, only two of the four conjugates showed substantial bias toward Gα_q_ activation over β-arrestin recruitment. The factors that lead to some nanobody–ligand conjugates to exhibit highly biased agonism (see PTH1 receptors and GLP-1 receptor sections) with others showing more limited pathway specificity (NK_1_ receptors and AT_1_ receptors) require further investigations.

To further interrogate the mechanism of receptor engagement by the bitopic conjugates, radioligand competition assays were performed (Braga Emidio et al., 2023). Surprisingly, a lead bitopic conjugate displaced only ~20% of radio-labeled orthosteric ligand at saturating concentrations, despite retaining full downstream signalling activity. This behaviour from nanobody–ligand conjugates may result from non-concurrent binding of the nanobody and ligand at their binding sites on the receptor. Alternatively, high-affinity binding of the nanobody to its epitope could impose constraints that restrict full occupancy of the orthosteric pocket by the tethered ligand. This unexpected pharmacological profile suggests that for nanobody–ligand conjugates, nanobody docking causes the peptide ligand to adopt a non-canonical mode of orthosteric binding, which may contribute to the unusual signalling profiles observed. Whether such non-canonical modes of engagement are operational for other GPCRs targeted by antibody- or nanobody–ligand conjugates will be an interesting question for further investigation.

### 4.2 | Small-molecule bitopic conjugates

#### 4.2.1 | Adenosine A_2A_ receptors

Many GPCRs do not respond to peptides, but rather to small molecule ligands. Unlike the peptide conjugates described above, the synthesis of antibody- (or nanobody-) small-molecule conjugates requires the development of bespoke synthetic chemistry to facilitate the installation of linkers that do not decrease small molecule bioactivity. This challenge was addressed in the design of conjugates that target A_2A_ receptors, a prototypical class A GPCR (Sachdev, Roy, & Cheloha, 2025a). A_2A_ receptor signalling is involved in a variety of physiological processes and disease states (Jacobson et al., 2022). CGS21680 (CGS), an A_2A_ receptor agonist with extensively characterized pharmacology, was chosen as the small-molecule building block for synthesis of nanobody–ligand conjugates. A carboxylic acid moiety on CGS that projects from the extracellular vestibule of A_2A_ receptors (Lebon et al., 2015) was used as a site for linkage with a nanobody to produce nanobody–CGS conjugates via click chemistry (Table 1). As described above for NK_1_ receptors, epitope tags were appended onto the A_2A_ receptor N-terminus, enabling nanobody engagement without perturbation of the receptor orthosteric site. Nanobody–CGS conjugates displayed potent and selective activation only at the receptor variants tagged with the cognate epitope tag. Nanobody–CGS conjugates induced more enduring cAMP responses than CGS alone. These findings suggest that targeting small molecule binding GPCRs with nanobody–ligand conjugates offers a path for potent, selective and prolonged modulation of receptor activity.

### 4.3 | Bitopic nanobody–nanobody biologic conjugates

#### 4.3.1 | Metabotropic glutamate receptors (mGluRs)

**FIGURE 5:**
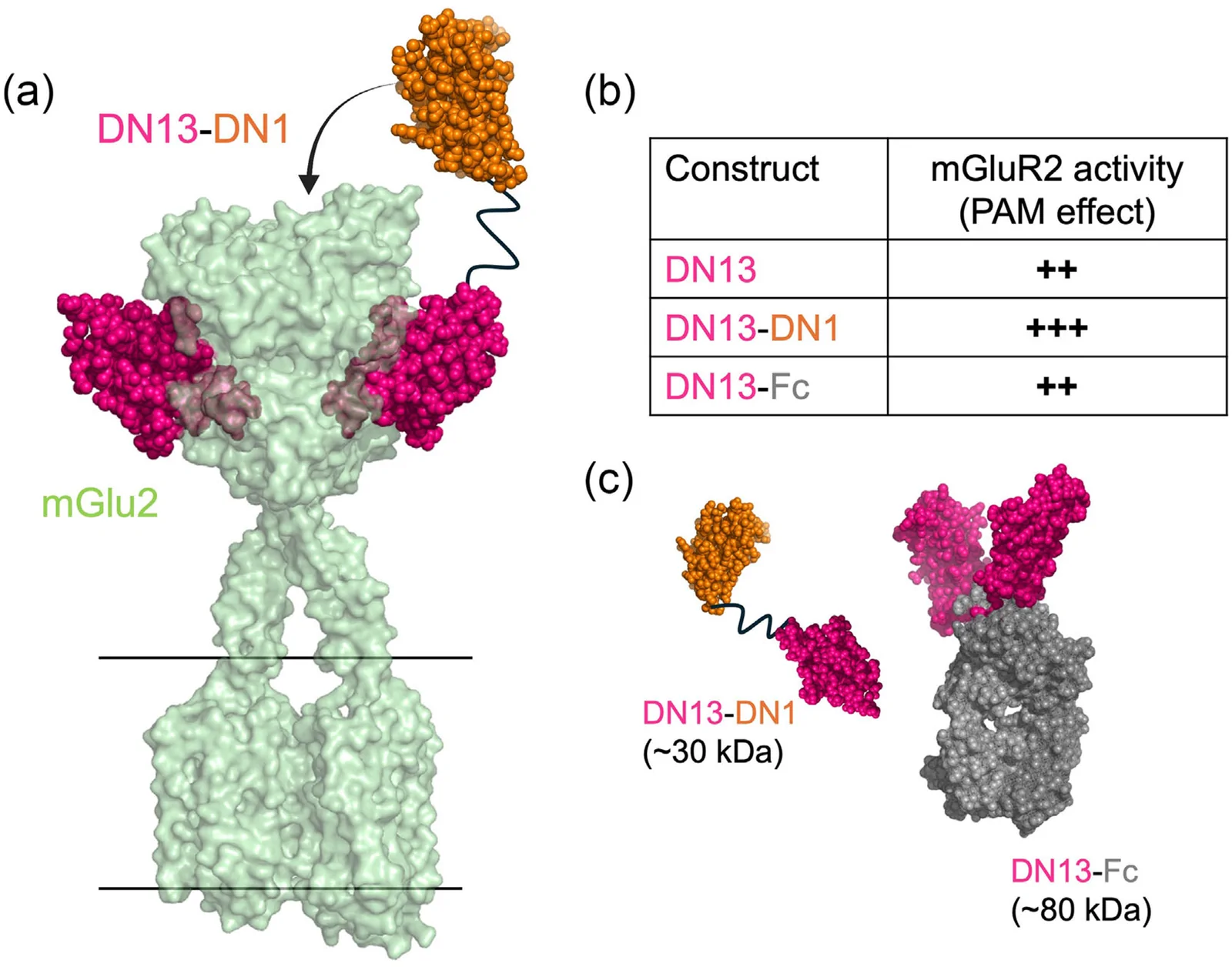
Bitopic nanobody–nanobody constructs targeting the mGlu_2_ receptor. (A) Schematic of a bitopic nanobody construct (DN13–DN1) engaging mGlu_2_ receptors. This structure is based on experimental data (mGlu2 bound to DN13, PDB 7E9G). No experimental structure is available for DN1 in complex with mGlu_2_ receptors. DN13 and DN1 nanobodies are connected using the hinge region from llama heavy-chain antibody (black line). (B) Summary of the pharmacological activity of bitopic constructs in a mGlu_2_ receptor (mGluR2) signalling assay (G_q_–IP_1_ accumulation) in the presence of an orthosteric agonist (LY379268). ‘+**’** symbols denote the relative levels of enhancement of IP_1_ accumulation by mGlu_2_ receptors by indicated constructs. (C) Schematic comparing the relative sizes of DN13–DN1 and DN13–Fc constructs.

mGlu receptors are a subgroup of class C GPCRs that respond to the excitatory neurotransmitter glutamate. Their signalling activity has been implicated in several neuropsychiatric and neurodegenerative disorders, prompting efforts to develop more selective modulators (Crupi et al., 2019). A series of nanobodies has been developed to selectively modulate different mGlu receptor subtypes. This series includes DN1 (a neutral binder for mGlu_2_ receptors) (Scholler et al., 2017), DN10 (a positive allosteric modulator [PAM] and agonist for mGlu_2_ receptors) (Scholler et al., 2017), DN13 (a PAM for mGlu_2_ receptors) (Scholler et al., 2017), DN45 (an agonist for mGlu_4_ receptors and a PAM for the mGlu_2_–mGlu_4_ heterodimer) (Haubrich et al., 2021), DN42 (mGlu_4_ receptor binder) (Meng et al., 2022) and Nb43 (mGlu_5_ receptor PAM) (Koehl et al., 2019). A bitopic nanobody construct (DN13–DN1) was engineered by genetically fusing two nanobodies that target mGlu_2_ receptors at different sites (Table 1 and Figure 5A) (Oosterlaken et al., 2025). DN13–DN1 demonstrated increased binding to mGlu_2_ receptors and a corresponding increase in PAM activity compared to monovalent DN13, implying avidity effects (Figure 5B). This bitopic construct was subsequently tested in a mouse model of NMDA receptor hypofunction and schizophrenia. Systemically dosed DN13–DN1 restored cognitive performance for up to 7-day post-administration, far exceeding the duration of action of small molecules targeting the same receptor (Oosterlaken et al., 2025).

The performance of the DN1-DN13 construct was unexpected given the localization of mGlu_2_ receptors in the CNS, which is thought to be protected from systemically administered protein agents by the blood-brain-barrier (BBB) (Oosterlaken et al., 2025). A DN13 Fc-fusion, which is substantially larger the DN13–DN1 construct (Figure 5C), showed strong activity in cell-based assays, but it failed to produce behavioural effects in the same mouse model. This difference may be due to limited BBB penetration by the IgG-format construct, perhaps related to its large size. A non-exclusive alternative is that the improved activity observed with the DN13–DN1 construct may relate to an enhanced targeting capacity driven by binding of the high-affinity of neutral binder DN1. These findings open new avenues for the development of multivalent nanobody constructs for addressing dysfunction within the CNS.

#### 4.3.2 | Chemokine receptor CXCR2

The chemokine receptor system operates across immune and nonimmune cells and contributes to a wide range of physiological and pathological processes, including cancer, inflammation, autoimmunity, metabolic and neurological disorders (Hughes & Nibbs, 2018). Composed of numerous receptor–ligand pairs with a high degree of redundancy and degeneracy, this system presents a major challenge for the development of selective targeted therapeutics (Kleist et al., 2025). Here, we focus on bivalent constructs targeting CXCR2. A panel of nanobodies targeting the extracellular face of human CXCR2 were characterized (Bradley et al., 2015). These nanobodies fell into two groups based on epitope specificity: group 1 nanobodies bound the N-terminal region of CXCR2 (such as 127D1), while members of group 2 recognized conformational epitopes at the extracellular loops of the TMD (such as 163E3). Group 2 nanobodies showed stronger inverse agonist efficacy and competitive antagonism, whereas Group 1 nanobodies showed weaker inverse agonism without exhibiting competitive inhibition.

Whether combining these nanobodies into bivalent or bitopic formats could improve inhibitory activity at the receptor was also tested (Bradley et al., 2015). Constructs composed of either two nanobodies from the same group (bivalent) or a combination of nanobodies from the two groups (biparatopic) were compared. Functional studies revealed that a bivalent construct retained a similar profile to its monovalent counterpart, whereas the biparatopic nanobody (127D1–163E3, Table 1) showed enhanced inhibition. To address whether biparatopic engagement occurred on a single receptor or across a pair of protomers, a dual-receptor system was used. Two CXCR2 variants, each mutated to ablate one of the nanobody-binding epitopes, were co-expressed. These experiments suggested that biparatopic nanobody constructs bridge adjacent receptor protomers, with analogy to findings with nanobody–ligand conjugates targeting PTH1 receptors (Sachdev et al., 2024). Bivalent nanobody-targeting has been also applied to other chemokine receptors, including CXCR4 (Jähnichen et al., 2010), underscoring the utility of this approach for modulating chemokine receptor pharmacology.

## 5 | NEW TOOLS TO EXPAND THE POOL OF ACCESSIBLE TARGETS: NANOBODY DISCOVERY, DESIGN AND ENGINEERING

Despite recent advances, only a fraction of GPCRs have validated nanobody binders, with even fewer examples of receptors bound by nanobodies at extracellular epitopes (Salom et al., 2024). New tools and approaches are needed to accelerate the discovery of binders for novel targets and epitopes. Traditionally, the development of conventional antibodies (and nanobodies) typically relies on animal immunization, in which hosts (such as camelids) are repeatedly exposed to a purified antigen to produce a humoral response (Pardon et al., 2014). Antigen-specific binders are then recovered by cloning nucleic acid sequences recovered from B cell clones, followed by enrichment of binding clones through phage display or related selection platforms. This multistep process, if successful, typically takes more than 6 months to yield a high-affinity nanobody binders and is associated with several limitations such as high cost, animal-welfare concerns and immunological barriers that impede generation of binders against antigens with high homology to host proteins. These limitations can be addressed using *in vitro* synthetic discovery platforms (Muyldermans, 2021). Synthetic nanobody libraries, comprising predesigned repertoires of nanobody sequences built on humanized or camelid frameworks, enable rapid binder discovery. Such libraries can be designed to incorporate specialized design elements, such as elongated CDRs to facilitate engagement of concave epitopes (Zimmermann et al., 2018). Strategies using synthetic libraries have enabled the identification of nanobodies against difficult targets, including GPCRs (Krohl et al., 2023). Screening synthetic libraries using platforms such as yeast display can enable binder identification within weeks (McMahon et al., 2018); however, other challenges exist. Access to synthetic libraries is often restricted and binders extracted from these libraries may exhibit polyreactivity, suboptimal biophysical behaviour or reduced thermostability, when compared with those recovered from animal immunization campaigns.

Another development accelerating nanobody discovery centres on the emergence of computational design and screening approaches. Early efforts described CDR loop grafting techniques, in which CDRs from existing antibody structures were transplanted onto nanobody scaffolds to explore new binding surfaces (Lippow et al., 2007). Next, docking algorithms were used to position these grafted or designed variants onto static models and score the resulting interfaces for complementarity and stability (Liu et al., 2017; Sormanni et al., 2015). More recently, deep-learning–based structure prediction tools such as AlphaFold and RoseTTAFold have been integrated with generative design engines, including RFdiffusion, to diversity and expand binder design (Abramson et al., 2024; Adolf-Bryfogle et al., 2018; Harvey et al., 2025; Watson et al., 2023). Such computational pipelines can now facilitate the identification of binders (nanobody or otherwise) against difficult-to-target or poorly immunogenic GPCR epitopes. Deep-learning–guided design has already delivered nanobodies with nanomolar affinity for GPCRs such as MRGPRX1, CXCR4, GLP-1 receptors, GIP receptors, GCG receptors and CGRP receptors (Muratspahić et al., 2025). De novo computational strategies complement synthetic libraries and display technologies, expanding the toolkit available for engineering the next-generation of bitopic GPCR conjugates.

## 6 | CONCLUSIONS, CHALLENGES AND FUTURE DIRECTIONS

Multivalent conjugates incorporating antibodies or other biological affinity reagents regularly exhibit advantages over small molecule-based approaches (Till et al., 2025). Their modular architecture allows straightforward engineering of bitopic constructs, through genetic fusion or site-specific labelling chemistries, to achieve highly selective receptor engagement. Empirically, the composition and length of linkers used in antibody- (or nanobody-) multivalent conjugates seems to be more tolerant to variation in linker composition than corresponding small molecule-based multivalent compounds. Bioconjugate-based bitopic conjugates can also be readily applied to engage receptor assemblies. Such efforts demand selectivity in engaging the target assembly over any one individual component, which has been demonstrated for nanobody–ligand bioconjugates (Sachdev, Roy, & Cheloha, 2025a). Similar selectivity with small-molecule bitopic ligands has been challenging to realize. Achieving activation dependent on co-expression of two targets may for allow cell type-selective pharmacology and logic-gated signalling (Sachdev, Roy, & Cheloha, 2025a). Such approaches may be valuable in targeting ligands with widely expressed receptors to cell types or tissues of choice (for example, within a tumour niche), creating opportunities for therapeutic ligands or payloads to act preferentially in cells that co-express the relevant receptor assembly while limiting activity at the same receptor in other tissues.

The characteristics of nanobody-based conjugates highlighted above, such as small size, avidity effects and tunable linker chemistry, offer exciting prospects for therapeutic development. Tissue- or cell-specific activity may enable the realization of therapeutic benefits while avoiding on-target, off-tissue side effects. The small size of nanobodies may be useful for enhancing tissue penetration (relative to full-size antibodies), which is valuable for engaging densely packed tissues such as tumours. Linking small molecules, which often have poor, variable and/or unpredictable pharmacokinetic behaviour, to nanobodies may also promote extended durations of circulation in the bloodstream.

These same design principles described above are applicable for the generation of modalities with biological properties that extend beyond receptor activation or blockade. For example, antibody-based bitopic conjugates have been used to target GPCRs for degradation or relocalization (Korona & Itzhaki, 2024). Dual targeting of GPCRs and membrane-associated E3 ligases or receptors that undergo constitutive internalization can alter receptor function and pharmacology (Rhee et al., 2025). Such bitopic constructs may be useful in eliminating receptors showing constitutive activity or abnormal downstream pathway engagement in pathological states (Portales-Castillo et al., 2023), providing an alternative to traditional inhibitor design. Related bitopic formats can also be adapted to induce proximity between two cell types, generating highly localized T-cell or NK-cell engagers that target GPCR-expressing cells in disease (Hutchings et al., 2026; Pillarisetti et al., 2020).

While multivalent bioconjugates hold considerable promise, their clinical translation faces practical hurdles. The rapid renal clearance of small antibody fragments (such as nanobodies) from the bloodstream results in transient physiological responses. This shortcoming can be addressed with engineering approaches to increase the apparent molecular weight of bioconjugates via modification with large PEG polymers (Rashidian et al., 2016) or linkage to moieties that bind serum albumin (Glassman et al., 2020). The limited ability of proteinsized bioconjugates to cross the blood–brain barrier poses challenges for intervening in CNS pharmacology. Linking bioconjugates to moieties that bind endogenous transporters such as the transferrin receptor may enable active shuttling across the blood–brain barrier (Esparza et al., 2023). As these practical challenges are addressed, antibody-based bioconjugates will provide new tools and clinical candidates for mechanism-driven modulation of GPCR function that moves beyond conventional activation or blockade.

### 6.1 | Nomenclature of targets and ligands

Key protein targets and ligands in this article are hyperlinked to corresponding entries in the IUPHAR/BPS Guide to PHARMACOLOGY (http://www.guidetopharmacology.org) and are permanently archived in the Concise Guide to PHARMACOLOGY 2025/26 (Alexander, Davenport, et al., 2025; Alexander, Fabbro, et al., 2025; Alexander, Gibb, et al., 2025).

## Figures and Tables

**TABLE 1:** List of antibody (Ab)- or nanobody (Nb)-based multivalent conjugates targeting GPCRs.

GPCR	Bitopic biologic conjugate	Ab/Nb epitope, isotype (pharmacology)	Conjugation strategy	Pharmacology	References
Peptide-biologic conjugates					
GLP-1 receptor (GLP1R)/GIP receptor (GIPR)	Ab_GIPR_–GLP1 (AMG133)	GIPR-ECD, IgG (antagonist)	Ab-Cys + bromoacetyl-GLP1 reaction	cAMP, β-arr, co-internalization, active in vivo	(Lu et al., 2021)
	Nb_GLP1R_-GLP1-mut	GLP1R-ECD, Nb (neutral)	SPAAC reaction	cAMP, no β-arr, not evaluated in vivo	(Sachdev et al., 2024)
	GLP-1 IgG (TB59–2)	GLP1R, IgG (neutral)	Genetic fusion	cAMP > β-arr, active in vivo	(Liu et al., 2021)
	GLP-1 A8G–Nb_GLP1R_-Nb_albumin_ (Everestmab)	GLP1R ECD + albumin, Nb (unknown)	Genetic fusion	Not reported, active in vivo	(Pan et al., 2020)
PTH1 receptor (PTHR1)	Nb_PTHR1_-PTH(1–14) Nb_PTHR1_-PTH(1–11)-AND-Nb_PTHR1×2_-PTH (1–11)	PTHR1 ECD, Nb PTHR1 ECD (two distinct epitopes), Nb	SPAAC reaction SPAAC reaction	cAMP, β-arr agonist, active in vivo cAMP, NO β-arr or Gα_q_, not evaluated in vivo	(Cheloha, Fischer, et al., 2020) (Sachdev et al., 2024)
NK_1_ receptor epitope tags (6E, Alfa, BC2)	Nb-NKA; Nb-SP_6–11_	N-terminal epitope tags (ALFA, 6E, BC2) extracellular portion, Nbs	Sortase A–mediated ligation	Gα_s_ (cAMP), Gα_q_ and β-arr agonism; pathway selectivity, not evaluated in vivo	(Braga Emidio & Cheloha, 2024a)
AT_1_ receptor, epitope tag (6E)	Nb_6E_-AngII; Nb_6E_-TRV027; Nb_6E_-TRV055; Nb_6E_-TRV055	N-terminal epitope tag (6E) in extracellular portion, Nb	Sortase A–mediated ligation	Gα_q_, and β-arr agonism; pathway selectivity, not evaluated in vivo	(Braga Emidio et al., 2023)
Small-molecule bitopic conjugates					
A_2A_ receptor, epitope tags (6E, Alfa, BC2)	Nb-CGS	N-terminal epitope tags (ALFA, 6E, BC2) extracellular portion, Nbs	SPAAC reaction	Enhanced Gα_s_ (cAMP) agonism, not evaluated in vivo	(Sachdev, Roy, & Cheloha, 2025b)
Bitopic Nb-Nb Biologic Conjugates					
mGlu_2_	Biparatopic Nb dimer (DN13-DN1)	mGluR2 ECD, (VFT domain; two distinct epitopes), Nbs	Genetic fusion	Enhanced G protein agonism, active in vivo (rescued behavioural deficits)	(Oosterlaken et al., 2025)
CXCR2	Biparatopic Nb dimer (Nb127D1–Nb163E3)	CXCR2 ECD (N-terminal domain) + transmembrane domain epitopes, Nbs	Genetic fusion	Inverse agonism of G protein signalling, enhanced competitive antagonism, not evaluated in vivo	(Bradley et al., 2015)
CXCR4	Biparatopic Nb dimer (238D2–238D4)	CXCR4 Extracellular loop 2 (overlapping epitopes), Nbs	Genetic fusion	Inverse agonism of G protein signalling, active in vivo (induced stem cell mobilization)	(Jähnichen et al., 2010)

## Data Availability

Data sharing is not applicable to this article because no new data were created or analysed in this study.

## What is already known?

- Bitopic small molecule-based ligands engage multiple pockets on GPCRs to enable selective receptor engagement.
- Emerging antibody and nanobody modalities engage unique GPCR epitopes and enable highly selective targeting.

## What does this study add?

- Case studies of antibody-peptide, nanobody–nanobody and small molecule–nanobody conjugates demonstrating multivalent GPCR engagement.
- Advances in bioconjugation chemistry and antibody engineering to control construct topology, homogeneity and pharmacological properties.

## What is the clinical significance?

- Multivalent conjugates may improve therapeutic indices by directing ligands to specific biological niches to reduce side effects.
- Modularity enables programmable ligand delivery and pathway-selective GPCR signalling not readily achievable with conventional ligands.

## Abbreviations:

Ab: antibody
ADC: antibody–drug conjugate
Aib: α-aminoisobutyric acid
AngII: angiotensin II
BBB: blood–brain barrier
CDR: complementarity-determining region
CuAAC: copper-catalysed azide–alkyne cycloaddition
DARPins: designed ankyrin repeat proteins
DOTA: 1,4,7,10-tetraazacyclododecane-1,4,7,10-tetraacetic acid
ECD: extracellular domain
Fc: fragment crystallizable region
FDA: Food and Drug Administration
Her2: human epidermal growth factor receptor 2
IgG: immunoglobulin G
Nb: nanobody
NKA: neurokinin A
PAM: positive allosteric modulator
PDB: Protein Data Bank
PEG: polyethylene glycol
PTH: parathyroid hormone
SP6–11: substance P residues 6–11
SPAAC: strain-promoted azide–alkyne cycloaddition
SrtA: sortase A
SuFEx: sulphur–fluoride exchange
TMD: transmembrane domain
VFT: Venus flytrap domain
VHH: variable domain of heavy-chain-only antibody
